# High-altitude hypoxia alters the developmental trajectory of working memory in Tibetan children

**DOI:** 10.3389/fnins.2026.1843212

**Published:** 2026-07-22

**Authors:** Xiaolin Yang, Chao Fu, Yongkui Zhao, Wenjing Di, Chun Zheng

**Affiliations:** 1 School of Teacher Education, Qinghai Minzu University, Xining, China; 2 School of Education, Qinghai Normal University, Xining, China; 3 School of Physical Education, Qinghai Normal University, Xining, China

**Keywords:** blood pressure, developmental trajectory, high-altitude hypoxia, Tibetan children, working memory

## Abstract

**Background:**

Verbal working memory (WM) undergoes rapid development during school age, yet the impact of high-altitude hypoxia on this trajectory remains unclear. We propose two distinct models: the simple additive model, which posits that hypoxia imposes a uniform impairment independent of age, preserving developmental gains; the developmental trajectory reconfiguration model, which posits that hypoxia alters the typical age-related progression of WM. To test these models and explore related physiological factors, we examined WM performance and blood pressure (BP) in school-aged children across different altitudes.

**Methods:**

A 2 × 3 between-subjects design was used, involving 535 Zang (Tibetan) students (273 fourth graders, 262 eighth graders) from three altitudes (2,200, 3,200, and 4,200 m) in Qinghai, China. WM was assessed using the Digit Span Backward (DSB) task, and BP was measured with a wrist-type electronic sphygmomanometer.

**Results:**

We found a significant main effect of altitude on WM [$F_{\left(2,529\right)}=34.18,p<0.001,\eta_{\text{p}}^{2}=0.11$], indicating that WM in children declined markedly at altitudes around 4,200m. A significant grade-by-altitude interaction was also observed [$F_{\left(2,529\right)}=5.38,p=0.005,\eta_{\text{p}}^{2}=0.02$]. Model comparison favored the developmental trajectory restructuring model over the simple additive model [LRT:χ2(2)=10.77,p=0.005], supporting the hypothesis that high-altitude hypoxia alters the WM developmental trajectory. Further moderated regression analysis revealed a significant three-way interaction among grade, diastolic blood pressure (DBP), and altitude [Δ*R*^2^ = 0.01, *F*_(2,512)_ = 3.73, *p* = 0.025], suggesting that the moderating effect of DBP on WM development is altitude-dependent. Simple slope analysis showed that at 2,200 and 3,200 m, eighth graders outperformed fourth graders in WM only when DBP was at low or mean levels; at 4,200m, no significant grade differences were observed regardless of DBP level.

**Conclusion:**

High-altitude hypoxia alters the developmental trajectory of verbal WM in Tibetan children, as evidenced by the disappearance of the typical grade advantage at higher altitudes and the altitude-dependent moderating role of DBP. These findings support the developmental trajectory reconfiguration model and highlight the physiological relevance of blood pressure in altitude-related cognitive development.

## Introduction

Working memory (WM), a core component of executive function, refers to the dynamic cognitive process by which individuals temporarily encode, maintain, and manipulate information. It serves as the foundation for higher-order cognitive processes such as information integration, reasoning, and problem-solving (Baddeley, 1998). The school-age period (4–15 years) represents a critical developmental window for WM, during which its functional maturation directly determines a child’s learning capacity, academic achievement, and long-term cognitive potential. Indeed, WM has been shown to effectively predict individual performance across various learning contexts, including mathematical accuracy, reading comprehension, and classroom engagement (Alloway and Alloway, 2010; Fry and Hale, 1996; Giedd et al., 1999; Swanson and Alloway, 2012; Swanson et al., 2009).

Globally, approximately 140 million people reside permanently at altitudes above 2,500 m, including a substantial population of school-aged children in the midst of rapid development (León-Velarde et al., 2005). Plateau regions are characterized by chronic hypobaric hypoxia, where hypoxic stress intensifies alongside rising elevation. Given that hypoxia significantly modulates central nervous system function (Li and Wang, 2022; Zhang and Zhang, 2022), it may disrupt the normal developmental trajectory of WM, thereby limiting cognitive function and ultimately affecting learning abilities and overall development. Therefore, a systematic investigation into the developmental characteristics and underlying regulatory mechanisms of WM in school-aged children living in moderate and high-altitude hypoxic environments is of considerable importance.

Extant research has established that WM development spans from infancy to early adulthood, with the school-age period representing a critical stage of rapid functional maturation and structural refinement. During infancy (0–3 years), WM abilities improve progressively with age, accompanied by the gradual emergence of basic mnemonic strategies (Ulug and Özçelik, 2024): infants aged 0–12 months can perform fundamental memory operations such as temporal sequence learning and auditory cue tracking (Applin and Kibbe, 2019; de Hevia et al., 2020; Guillory and Kaldy, 2019); toddlers aged 13–24 months develop the ability to utilize perceptual contrasts and verbal counting to support memory (Wang and Feigenson, 2019; Zosh and Feigenson, 2015); and children aged 25–36 months can complete more complex memory tasks, including multiple-identity tracking and symbolic learning (Cheng et al., 2019; Choi et al., 2021; Kibbe and Applin, 2022). Upon entering the school-age years, WM enters a phase of accelerated development. In a large-scale study of over 700 children aged 4–15 years, Gathercole et al. (2004) found that the basic structure of WM is established by at least age 6, with its functional capacity continuing to expand significantly throughout elementary and middle school. Specifically, the ability to update object locations based on changes in occlusion improves with age in children aged 4–7 years (Cheng and Kibbe, 2022). Meanwhile, verbal WM capacity, as measured by digit span backward (DSB) tasks, nearly doubles between ages 5 and 11.5 years, increasing from an average of two digits at age 5.5 to four digits at age 10 (Finch, 2019; Reynolds et al., 2022). At the neural level, children aged 9–13 years have already established a core WM network that highly overlaps with that of adults. However, their pattern of brain activation undergoes a transition from a diffuse pattern to a more focused and efficient one, reflecting the maturation of cognitive function (Brahmbhatt et al., 2010; Geier et al., 2009). Notably, different WM functions mature at different times. Simple maintenance functions develop relatively early, whereas more complex functions involving monitoring, updating, and attentional focus switching follow a protracted developmental trajectory that continues through adolescence and into early adulthood (Alloway and Alloway, 2013; Ferguson et al., 2021; Lendínez et al., 2015). For instance, the period between ages 5 and 11 represents a phase of rapid improvement in visual maintenance, while spatial maintenance, which relies on complex sequential representation and executive control, develops more slowly and does not reach maturity until young adulthood (Hamilton et al., 2003). Additionally, children aged 10–12 years achieve mature performance on simple memory updating tasks (1-back) but show a linear developmental trend on high-load 2-back tasks requiring interference control, a trajectory that extends from childhood into young adulthood (Brahmbhatt et al., 2010; Schleepen and Jonkman, 2009). Overall, WM functions generally peak in adulthood. Visual WM capacity increases from one to two items in infancy to four to six items in young adulthood, reaching its peak around age 30 (Alloway and Alloway, 2013; Simmering, 2016).

Hypoxia, as a critical environmental stressor, exerts significant effects on the structure and function of the central nervous system (Chen et al., 2023; Zhang and Zhang, 2022). Given that WM is highly sensitive to hypoxia, its developmental trajectory is particularly vulnerable to hypoxic disruption. Existing evidence indicates that hypoxic exposure during early life, whether prenatal, perinatal, or postnatal, can cause long-lasting or even irreversible WM impairments. In rats, even brief prenatal hypoxia can lead to hippocampal neuron loss and significant WM deficits in adulthood (Golan et al., 2004, 2009). Hypoxic-ischemic encephalopathy (HIE) resulting from perinatal hypoxia leads to persistent WM impairments in affected children during school age and adolescence, with performance lagging significantly behind that of healthy peers (Halpin et al., 2022; Hayes et al., 2018; Marlow et al., 2005). Postnatal hypoxia, as demonstrated in both animal models and clinical studies, also disrupts hippocampal function and subsequently impairs WM performance (Balduini et al., 2000; Cooper et al., 2015; Parmar et al., 2024; Vannucci and Vannucci, 2005).

It is important to note that the early-life hypoxia described above is primarily pathological in nature. In contrast, school-aged children living at high altitudes are exposed to chronic, sustained environmental hypoxia, which differs fundamentally in terms of intensity, duration, and mode of action. Currently, research directly examining the impact of high-altitude hypoxia on WM development in school-aged children remains scarce. Existing cognitive studies conducted at high altitudes have focused primarily on adults, and these have consistently demonstrated a significant altitude-dependent effect of hypoxia on adult WM. Specifically, within the altitude range of 1,500–3,000 m, WM performance remains generally stable: short-term exposure to altitudes of 1,630, 2,590, and 3,800 m did not result in significant WM impairments compared to baseline levels at sea level (Frost et al., 2021; Latshang et al., 2013); long-term residents at 2,260 m showed no WM deficits, with the brain employing neural compensatory mechanisms to offset the mild hypoxic stress (Chen et al., 2022; Zhang et al., 2011). Further evidence suggests that when altitude increases to the 3,000–4,000 m range, WM impairments begin to emerge, with patterns varying between natives and migrants. In healthy young adults who were born at low altitude and migrated to 3,680 m for three years, performance on high-load verbal and spatial 2-back tasks showed decreased accuracy and prolonged reaction times (Ma H. et al., 2020; Ma et al., 2019). Among native Tibetan residents at 3,680 m, spatial WM remained intact, whereas verbal WM showed a reversible deficit that gradually recovered after relocation to low altitude (Li et al., 2026). In Han Chinese adults born and raised at altitudes between 2,616 and 4,200 m, spatial WM was maintained through neural compensation, whereas verbal WM was directly impaired (Yan X. et al., 2011; Yan X. A. et al., 2011). Above 4,000 m, neural compensatory mechanisms appear to reach their limit, and WM function shows a marked decline. In native Tibetans at 4,200 m, electroencephalographic activity in the delta and theta bands (biomarkers of WM impairment) was significantly reduced (Ma H.-L. et al., 2020). Studies using simulated hypoxia at 4,500 m, field exposure at 5,160 m, and simulated altitudes of 5,334 and 7,620 m have consistently confirmed significant WM declines in adults (Asmaro et al., 2013; Bliemsrieder et al., 2022; Lefferts et al., 2019; Williams et al., 2019). Collectively, these findings suggest that a potential altitude turning point for the transition from compensation to significant decline in adult WM may exist somewhere between 3,000 and 4,200 m.

The altitude-dependent effects of high-altitude hypoxia on WM are closely linked to the physiological compensatory mechanisms activated in response to hypoxic stress, with changes in blood pressure (BP) serving as one of the key compensatory pathways (Lang et al., 2021). Acute hypoxia initially reduces arterial oxygen saturation, which in turn triggers a hypoxic ventilatory response and sympathetic activation. These processes increase heart rate, enhance myocardial contractility, and elevate arterial BP, thereby compensating for insufficient tissue oxygen supply, a fundamental physiological adaptation to high-altitude hypoxia (Mitchell et al., 2018; Querido et al., 2011; Whayne, 2014). BP changes also exhibit a clear altitude-dependent pattern. Even moderate altitudes of approximately 2,000 m can induce a slight but significant elevation in 24-h BP among healthy individuals (Torlasco et al., 2020). At altitudes between 3,200 and 3,700 m, BP elevations become more pronounced, with a greater magnitude of increase observed in hypertensive patients compared to healthy individuals (Bilo et al., 2015; Parati et al., 2014; Rao et al., 2015). Above 4,000 m, the BP-elevating effect becomes even more prominent (Bilo et al., 2011; Parati et al., 2014; Rao et al., 2015). Following prolonged high-altitude exposure, healthy lowlanders may exhibit a gradual decrease in heart rate through acclimatization. However, 24-h BP remains persistently elevated above lowland baseline values (Bilo et al., 2011; Farias et al., 2013). Moreover, the nocturnal decline in BP is substantially blunted, resulting in a non-dipping BP pattern that does not fully recover (Bilo et al., 2011). High-altitude natives generally show increased arterial stiffness and chronically elevated BP levels (Hainsworth et al., 2007). In contrast, individuals with conditions such as chronic mountain sickness or hypertension exhibit even greater BP fluctuations following long-term high-altitude exposure, due to their impaired BP regulatory capacity (Hainsworth et al., 2007; Parati et al., 2014). Although individual variability in BP responses to high altitude exists (Keyes et al., 2017), BP changes, as a key physiological stress response to hypoxia, may play an important modulatory role in the WM developmental trajectory of rapidly growing children. However, the specific mechanisms underlying this association in children remain unclear.

Synthesizing the above evidence from cognitive and physiological research, four key premises can be established: (1) the period between 4 and 15 years is a critical window for rapid WM development, with capacity increasing steadily with age; (2) early-life hypoxic exposure significantly disrupts the normal WM developmental trajectory; (3) high-altitude hypoxia exerts an altitude-dependent effect on adult WM, with a potential turning point between 3,000 and 4,200 m; and (4) high-altitude hypoxia triggers compensatory BP responses that may modulate WM development. However, current research still has several important limitations. First, existing studies on high-altitude hypoxia and WM have focused primarily on adults, with limited research on school-aged children, leaving the pattern of hypoxic effects on WM development in this population unclear. Second, whether and how high-altitude hypoxia modulates WM development in children via BP compensatory responses remains unknown, with a notable lack of mechanistic investigation. Third, the development of WM in high-altitude children is likely shaped by the combined influence of developmental gains and altitude-related impairments, yet the interaction between these two factors (whether a simple additive effect or a trajectory restructuring) remains untested.

Accordingly, we propose two theoretical models. The “simple additive model” posits that developmental gains and altitude-related impairments independently and additively affect WM in high-altitude school-aged children. Under this model, WM shows significant age-related improvements across all altitudes. A uniform hypoxic deficit produces parallel developmental trajectories that shift downward with rising altitude. The “developmental trajectory restructuring model” posits an interaction between altitude and developmental stage, such that the hypoxic environment is associated with an alteration of the WM developmental trajectory. This alteration may be such that, when resources are limited, it could lead to a tendency for earlier stabilization, thereby attenuating or eliminating the cognitive advantages typically associated with increasing age at higher altitudes. The fundamental distinction between the two models lies in whether the altitude effect manifests as a quantitative change (i.e., unchanged developmental rate with reduced baseline) or a qualitative change (i.e., altered developmental rate or pattern).

To test these theoretical models and elucidate the developmental characteristics and related physiological factors of WM in school-aged children living in moderate and high-altitude hypoxic environments, the present study employed the DSB task, a classic measure of verbal WM capacity, to assess WM performance in fourth-grade (younger school-aged) and eighth-grade (older school-aged) children at three altitudes (2,200, 3,200, and 4,200 m). BP (systolic and diastolic) was measured concurrently. The specific objectives of this study were: (1) to characterize the developmental patterns of WM in school-aged children across altitudes and evaluate the applicability of the simple additive model versus the developmental trajectory restructuring model; and (2) to determine whether and how altitude-related BP changes modulate the developmental trajectory of WM in school-aged children, thereby exploring the physiological pathway through which high-altitude hypoxia influences WM development during childhood.

## Materials and methods

### Participants

A total of 535 Tibetan students residing in high-altitude regions participated in this study, comprising 273 fourth-grade (mean age: 11.23 ± 0.78 years) and 262 eighth-grade (mean age: 15.23 ± 0.65 years) participants. The students were recruited from primary and secondary schools in three counties of Qinghai Province, China: Guide County (2,200 m), Gangcha County (3,200 m), and Zhiduo County (4,200 m). All participants were native-born residents of their respective counties. To ensure balanced representation, three classes were selected from both primary and secondary schools within each county. The sex distribution was balanced across altitude levels and grade cohorts (Table 1).

**TABLE 1:** Sex distribution stratification by altitude and grade level.

Grade	Altitude	Male	Female	Total	χ^2^	*p*
Grade 4	Guide (2,200 m)	43	42	85	1.42	0.491
	Gangcha (3,200 m)	39	48	87		
	Zhiduo (4,200 m)	54	47	101		
	Total	136	137	273		
Grade 8	Guide (2,200 m)	46	48	94	0.47	0.792
	Gangcha (3,200 m)	44	47	91		
	Zhiduo (4,200 m)	41	36	77		
	Total	131	131	262		

To minimize potential confounding factors, the study design incorporated the following features. All participants were full-time boarding students. They received a unified boarding school diet, daily timetable, and routine physical activities managed by local school authorities, which partially balanced between-group differences in daily nutrition, sleep schedule, and physical fitness. The three surveyed counties (Guide, Gangcha, Zhiduo) are all traditional Tibetan inhabited areas with comparable socioeconomic development levels, local cultural customs, and resident lifestyles, which further minimized potential confounding effects induced by regional economic and social disparities.

The study protocol, including the consent procedures, was reviewed and approved by the Institutional Review Board of Qinghai Minzu University (Approval No. 2026-011). The IRB explicitly approved the use of verbal informed consent from legal guardians for this low-risk, school-based study, considering the practical constraints of the boarding school setting. Prior to recruitment, authorization was obtained from the school principals and homeroom teachers. Given the long-term boarding arrangement, school personnel and researchers fully explained the study purpose, procedures, potential discomforts, and anticipated benefits to guardians via class messaging groups and telephone calls. Verbal informed consent was then obtained from all guardians, and age-appropriate child assent was obtained from all participating students before testing began. All participants had normal or corrected-to-normal vision and no reported history of mental disorders, traumatic brain injury, or other major systemic diseases. Students were also informed of their right to withdraw from the study at any time without penalty or negative consequences.

The study employed a 2 (grade: Grade 4 vs. Grade 8) × 3 (altitude: 2,200 vs. 3,200 vs. 4,200 m) between-subjects design. *A priori* power analysis using G*Power 3.1 (Faul et al., 2009) determined that 400 participants would provide 95% power (α = 0.05, two-tailed) to detect medium effect sizes (Cohen’s *d* = 0.25) for primary group comparisons. Thus, the final sample size of 535 participants ensured adequate statistical power for all planned analyses.

### The digit span backward task

Verbal WM capacity was assessed using the DSB task (Hilbert et al., 2014), which was programmed and presented via E-Prime software (Version 2.0, Psychology Software Tools, Inc., Pittsburgh, PA, USA). Stimuli consisted of digits from 0 to 9, arranged in pseudorandom sequences ranging in length from 1 to 13 digits (sequences of 1 digit were not included in the scoring).

The task required the reverse-order recall of visually presented digit sequences. After viewing a sequence, participants typed the digits in reverse order into a response box (e.g., “2–6–9–5” for the sequence 5–9–6–2). No practice was provided, vocalization was prohibited, and participants self-initiated each trial with the spacebar.

**FIGURE 1:**
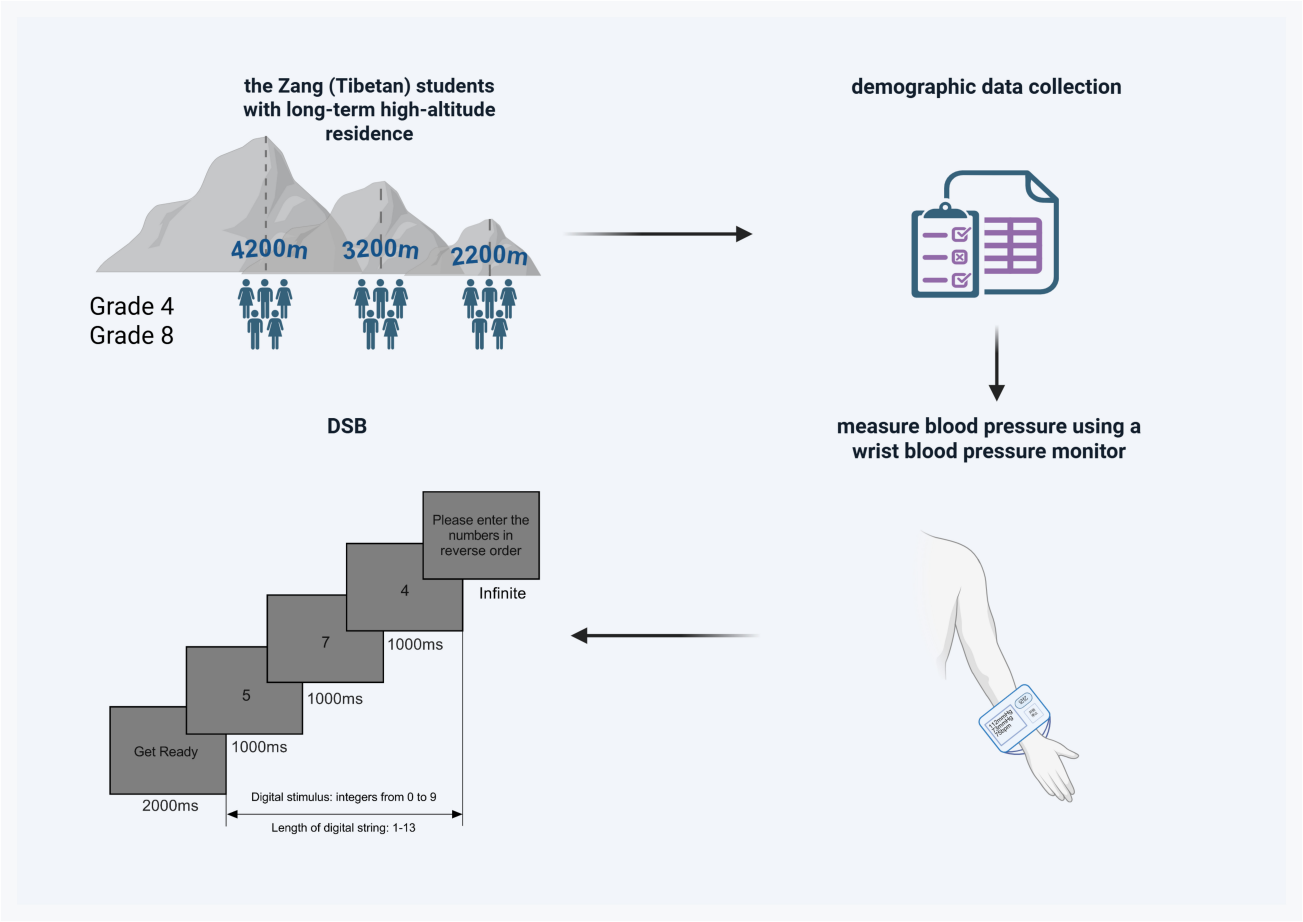
Experimental procedure. Following the acquisition of demographic information, systolic blood pressure (SBP) and diastolic blood pressure (DBP) were sequentially measured. Subsequently, working memory (WM) was assessed using the digit span backward task Created in BioRender. xiaolin, Y. (2026) https://BioRender.com/0upfv2m.

Each trial began with a “Get Ready” screen displayed for 2,000 ms to prompt participants to focus their attention. Digits were then presented sequentially in the center of the screen, each for 1,000 ms. After a sequence was presented, participants were required to type the digits in reverse order into a dialog box. If participants correctly recalled a sequence, the next sequence would be one digit longer. They had two attempts at each sequence length. The task was terminated if they failed both attempts at a given length. WM capacity score was defined as the longest sequence correctly recalled prior to termination. The stimulus sequence and presentation timing are illustrated in Figure 1.

### Measurement of blood pressure

BP was measured using a Yu Well wrist-type electronic sphygmomanometer (Model YE8800AR, Jiangsu Yuyue Medical Equipment Co., China). Before BP detection, all participants rested quietly in a seated position for 15 min to avoid acute exercise and mood-induced BP fluctuation. With the participant seated, the cuff was positioned on the wrist approximately 1.0–1.5 cm proximal to the base of the palm (about one finger’s width), ensuring its center was aligned at heart level. Prior to each measurement, the device automatically issued a verbal instruction (“Please do not move or speak”). Readings were recorded immediately upon completion. To ensure measurement consistency, the device was powered by a built-in rechargeable battery (rated 3.7 V). The measurement procedure involves automatic inflation of the cuff via a built-in pressure pump. During inflation, a semiconductor pressure sensor continuously detects and records pressure oscillations within the cuff. These oscillations are subsequently processed by an integrated algorithm to determine systolic blood pressure (SBP) and diastolic blood pressure (DBP). The technical specifications of the monitor are as follows: the pressure measurement range is 0–300 mmHg (0.0–40.0 kPa), with an accuracy of ± 3 mmHg ( ± 0.4 kPa).

### Experimental design and procedure

This study employed a 2 (grade: fourth vs. eighth) × 3 (altitude: 2,200, 3,200, 4,200 m) between-subjects design to investigate the developmental trajectory of WM in school-aged children living at high altitudes. The primary focus was on examining the interaction between altitude and grade, as well as analyzing whether BP modulates the developmental course of WM in these children.

To address these objectives, data collection was conducted in computer classrooms at each participating school. All tasks were administered on computers equipped with 21.5-inch displays (1920 × 1080 pixel resolution) and pre-installed E-Prime software. The experimental procedure is schematically illustrated in Figure 1. Participants first completed a basic information questionnaire collecting demographic details (school, grade, class, gender, and age). Next, their SBP and DBP were measured. Finally, they performed the DSB task.

### Data analysis

A total of 789 participants were initially recruited. During data preprocessing, 149 records were excluded due to missing data. Additionally, in accordance with the fundamental task requirements, a WM score of 0 or 1 was considered to reflect either a failure to comprehend the instructions or an inability to properly perform the task. These participants (*n* = 101) were excluded to ensure the validity of subsequent analyses. Extreme outliers, defined as values beyond ± 3 SD from the mean, were also removed. The final sample size for the DSB statistical analysis was 535. The collected physiological parameters included SBP and DBP. Outliers for SBP were identified and excluded according to established clinical guidelines and statistical principles. Based on international critical care standards, values were considered non-physiological if the corresponding estimated mean arterial pressure (MAP) fell below the critical threshold of 60–65 mmHg, which represents the lower limit for maintaining adequate organ perfusion (Evans et al., 2021; Hall, 2016). This cutoff was used solely to exclude biologically implausible extreme values due to measurement error, not for clinical diagnosis; the BP data serve only as population-level physiological indicators for statistical analysis. Using the standard formula (MAP ≈ DBP + 1/3 pulse pressure), a MAP of 60–65 mmHg corresponds to an approximate SBP range of 70–95 mmHg under typical clinical conditions (Evans et al., 2021), thereby establishing a physiologically grounded criterion for identifying critically low SBP outliers. Consequently, SBP outliers (8, 11, 11, 15, 16, 47, 53, 56, 66, 66, 67 mmHg) were excluded. After further excluding outliers beyond ± 3 SD from the mean, the final valid sample sizes were 516 for SBP and 524 for DBP.

A series of two-way analyses of variance (ANOVA) were conducted using SPSS to examine the effects of altitude (2,200, 3,200, 4,200 m) and grade level (fourth vs. eighth) on WM, SBP and DBP. Levene’s test was applied to test the homogeneity of variance assumption for each variable separately. When the assumption of homogeneity of variance was met, as indicated by a non-significant Levene’s test, standard ANOVA was interpreted. In cases of significant violation of homogeneity, heteroskedasticity-robust Type-III ANOVA was conducted via the Anova() function from the car package in R (with the argument white.adjust = TRUE) to ensure robust inference under heteroscedasticity. In the event of a significant interaction effect, simple effect analyses were conducted with LSD.

To test the two theoretical models (i.e., the simple additive model vs. the developmental trajectory restructuring model), we conducted hierarchical linear regression with likelihood ratio tests (LRT). The additive model (Model 1) included grade and altitude as predictors (WM ∼ Grade + Altitude), while the restructuring model (Model 2) additionally included the Grade × Altitude interaction term (formula: WM ∼ Grade + Altitude + Grade: Altitude, abbreviated as WM ∼ Grade * Altitude). Model comparison was performed using LRT, with a significant LRT (*p* < 0.05) indicating that the interaction model provided a superior fit. Additionally, Akaike Information Criterion (AIC) and Bayesian Information Criterion (BIC) were computed, with lower values indicating better model fit. The magnitude of ΔBIC was interpreted following Raftery’s (1995) criteria: | ΔBIC| < 2 indicates weak evidence, 2–6 positive evidence, 6–10 strong evidence, and > 10 very strong evidence for the model with the lower BIC value. All regression analyses were conducted using R with the lm() function, and LRT was performed using the lrtest() function from the lmtest package.

To examine whether BP moderates the grade-related development of WM, we conducted a moderated moderation analysis (i.e., three-way interaction) using the PROCESS macro (v3.5) for SPSS (Model 3) (Hayes et al., 2018). This model tests whether the moderating effect of BP (SBP or DBP) on the grade-WM association is itself moderated by altitude. Grade (fourth vs. eighth) served as the focal predictor, WM as the outcome, and SBP (or DBP) and altitude (2,200, 3,200, and 4,200 m) as the two moderators. To reduce multicollinearity and facilitate interpretation, SBP and DBP scores were mean-centered prior to analysis. Altitude, a multi-categorical variable, was dummy-coded with 3,200 m specified as the reference group. Statistical significance was set at *p* < 0.05 for all effects, with particular focus on the three-way interaction among grade, BP, and altitude. In the event of a significant three-way interaction, simple slopes analysis was conducted to examine the conditional effects of grade on WM at low, mean, and high levels of SBP or DBP (mean ± 1 SD) for each altitude condition.

## Results

### Interactive effects of altitude and grade on verbal working memory

**FIGURE 2:**
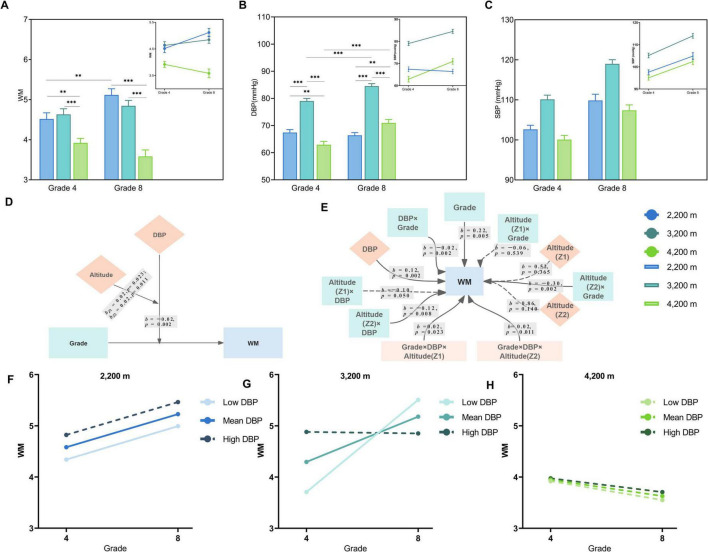
Altitude, grade, and diastolic blood pressure interact to shape working memory. Data are presented as mean ± SEM. Altitude-by-grade interactions. **(A)** Working memory (WM): Performance at 2,200 and 3,200 m was superior to that at 4,200 m in both grades. At 2,200 m, eighth graders outperformed fourth graders. **(B)** Diastolic blood pressure (DBP): Eighth graders exhibited higher DBP than fourth graders at 3,200 and 4,200 m. Within-grade comparisons showed DBP levels of fourth graders: 3,200 m > 2,200 m > 4,200 m; eighth graders: 3,200 m > 4,200 m > 2,200 m. **(C)** Systolic blood pressure (SBP): Eighth graders consistently had higher SBP than fourth graders across all altitudes. In both grades, SBP was highest at 3,200 m (3,200 m > 2,200 m, 4,200 m). Moderated moderation analyses. **(D)** Conceptual diagram illustrating the moderated moderation model: the moderating effect of DBP on the relationship between grade and WM is itself moderated by altitude. **(E)** Path diagrams of the moderated regression models. Grade is the independent variable, WM is the dependent variable, and DBP and altitude are moderators. Solid lines indicate paths with statistically significant unstandardized regression coefficients (*b*, *p*s < 0.05). **(F–H)** Simple slope analyses examining grade differences in WM at each altitude (2,200, 3,200, and 4,200 m) as a function of DBP level (low, mean, high). At 2,200 m **(F)** and 3,200 m **(G)**, eighth graders showed significantly better WM performance than fourth graders at low and mean DBP levels (*p*s < 0.05), but not at high DBP. At 4,200 m **(H)**, no significant grade differences were observed at any DBP level (*p*s > 0.05). In panels **(F–H)**, DBP values are plotted at three levels: M–1 SD (low), M (mean), and M + 1 SD (high). Solid lines indicate simple slopes that are statistically significant at *p* < 0.05. Error bars indicate standard errors. *p < 0.05, **p < 0.01, ***p < 0.001.

A two-way ANOVA (altitude × grade) on WM revealed a significant main effect of altitude [$F_{\left(2,529\right)}=34.18,p<0.001,\eta_{\text{p}}^{2}=0.11$], a non-significant main effect of grade [$F_{\left(1,529\right)}=1.85,p=0.174,\eta_{\text{p}}^{2}=0.00$], and a significant interaction effect [$F_{\left(2,529\right)}=5.38,p=0.005,\eta_{\text{p}}^{2}=0.02$] (Figure 2A). Simple effects analyses revealed that, across both grade levels, WM scores in the 4,200 m group were significantly lower than those in both the 2,200 m (4th: 3.92 ± 1.14 vs. 4.52 ± 1.42, *p* = 0.003; 8th: 3.58 ± 1.43 vs. 5.12 ± 1.50, *p* < 0.001) and 3,200 m (4th: 3.92 ± 1.14 vs. 4.63 ± 1.30, *p* < 0.001; 8th: 3.58 ± 1.43 vs. 4.85 ± 1.29, *p* < 0.001) groups. No significant differences were observed between the 2,200 m and 3,200 m groups at either grade level (*p*s > 0.05). Furthermore, eighth graders demonstrated significantly higher WM than fourth graders only at 2,200 m (5.12 ± 1.50 vs. 4.52 ± 1.42, *p* = 0.003), with no significant grade differences detected at 3,200 m (4.85 ± 1.29 vs. 4.63 ± 1.30, *p* = 0.290) or 4,200 m (3.58 ± 1.43 vs. 3.92 ± 1.14, *p* = 0.099).

### Model comparison: simple additive vs. restructuring model

To test whether high-altitude hypoxia simply reduces WM performance (simple additive model: WM ∼ Grade + Altitude) or alters the developmental trajectory (developmental trajectory restructuring model: WM ∼ Grade × Altitude), we compared two hierarchical linear regression models using likelihood ratio tests and information criteria.

The additive model explained 11.39% of the variance in WM performance (*R*^2^ = 0.11), while the restructuring model including the Grade × Altitude interaction explained 13.16% of the variance (*R*^2^ = 0.13). The restructuring model provided a significantly better fit than the additive model [Δ⁢R2=0.02,LRT:χ2(2)=10.77,p=0.005]. Consistent with this, AIC values favored the restructuring model (1,844.55 vs. 1,851.32 for the additive model). Although BIC marginally favored the additive model (1,874.52 vs. 1,872.73, ΔBIC = 1.79), this difference is considered “weak” evidence according to Raftery’s (1995) criteria, and the significant likelihood ratio test supports the interaction model. These findings corroborate the significant Grade × Altitude interaction from the two-way ANOVA and provide formal support for the developmental trajectory restructuring model.

### Effects of altitude and grade on blood pressure

For DBP, the heteroskedasticity-robust ANOVA revealed significant main effects of altitude [*F*_(2,518)_ = 62.01, *p* < 0.001,η^2^ = 0.32] and a significant altitude × grade interaction [*F*_(2,518)_ = 8.78, *p* < 0.001,η^2^ = 0.02], while the main effect of grade was not significant [*F*_(1,518)_ = 0.47, *p* = 0.491,η^2^ = 0.03]. Simple effects analysis showed no significant difference between grades at 2,200 m (67.42 ± 9.76 vs. 66.39 ± 9.83mmHg, *p* = 0.507). At 3,200 m and 4,200 m, eighth graders exhibited a higher DBP than fourth graders (3,200m:84.52 ± 8.71 vs. 79.05 ± 8.74mmHg;4,200m:70.94 ± 10.80 vs. 62.92 ± 12.63mmHg, *p*s < 0.001). Altitude had differential effects on the two grades. For fourth graders, DBP was highest at 3,200 m, followed by 2,200 m and then 4,200 m (*p*s < 0.01). For eighth graders, DBP followed the order of 3,200 m > 4,200 m > 2,200 m (*p*s < 0.01) (Figure 2B).

For SBP, the heteroskedasticity-robust ANOVA revealed significant main effects of altitude [*F*_(2,510)_ = 24.16, *p* < 0.001,η^2^ = 0.14] and grade [*F*_(1,510)_ = 14.67, *p* < 0.001,η^2^ = 0.09], while the altitude × grade interaction was not significant [*F*_(2,510)_ = 0.33, *p* = 0.717,η^2^ = 0.00] (Figure 2C). *Post-hoc* comparisons indicated that SBP was highest at 3,200 m, significantly exceeding values at both 2,200 and 4,200 m (*p*s < 0.001). Additionally, SBP at 2,200 m was significantly higher than at 4,200 m (*p* = 0.041). Regarding the grade effect, eighth graders exhibited significantly higher SBP than fourth graders overall (*p* < 0.001).

### Altitude-dependent moderating role of blood pressure

A moderated moderation analysis (PROCESS Model 3) was conducted to examine the three-way interaction among altitude, SBP, and grade on WM. The overall model was significant, accounting for 14% of the variance in WM [*R*^2^ = 0.14, *F*_(11,504)_ = 7.30, *p* < 0.001]. The three-way interaction (altitude × SBP × grade) was not significant [Δ*R*^2^ = 0.00, *F*_(2,504)_ = 0.49, *p* = 0.615], indicating that the moderating effect of SBP on the grade–WM relationship did not vary by altitude. Examination of the interaction components revealed that neither the grade × SBP interaction at 3,200 m versus 2,200 m(*b* = 0.00, *SE* = 0.01, *t* = 0.36, *p* = 0.717, 95%*CI*[−0.01, 0.02]) nor at 3,200 versus 4,200 m (*b* = −0.00, SE = 0.01, *t* = −0.57, *p* = 0.566, 95% CI [−0.02, 0.01]) reached statistical significance. However, a significant two-way interaction between grade and altitude (3,200 m vs. 4,200 m) emerged (*b* = −0.18, SE = 0.09, *t* = −2.03, *p* = 0.043, 95% CI [−0.35,−0.01]), suggesting that the effect of grade on WM differed between these two altitudes. No other two-way interactions or main effects were significant (*p*s > 0.05) (see Supplementary material for full regression output).

A moderated moderation analysis (PROCESS Model 3) was conducted to examine the three-way interaction among altitude, DBP, and grade on WM. The overall model was significant, explaining 15% of the variance in WM [*R*^2^ = 0.15, *F*_(11,512)_ = 8.16, *p* < 0.001]. The three-way interaction (altitude × DBP × grade) was significant [Δ*R*^2^ = 0.01, *F*_(2,512)_ = 3.73, *p* = 0.025], indicating that the moderating effect of DBP on the grade–WM relationship varied by altitude. Examination of the interaction components revealed that the grade × DBP interaction was significantly moderated by altitude when comparing 3,200 versus 2,200 m (*b* = 0.02, SE = 0.01, *t* = 2.28, *p* = 0.023,95% CI [0.00,0.03]) and when comparing 3,200 versus 4,200 m (*b* = 0.02, SE = 0.01, *t* = 2.56, *p* = 0.011,95% CI [0.00, 0.03]) (see Supplementary material for full regression output and Figures 2D, E).

Simple slope analyses revealed distinct patterns across altitudes. At 2,200 and 3,200 m, grade positively predicted WM at low DBP (*b* = 0.16, SE = 0.07, *t* = 2.48, *p* = 0.014,95% CI [0.03,0.29];*b* = 0.45, SE = 0.14, *t* = 3.15, *p* = 0.002,95% CI [0.17,0.73]), and mean DBP (*b* = 0.16, SE = 0.06, *t* = 2.86, *p* = 0.005,95% CI [0.05,0.27];*b* = 0.22, SE = 0.08, *t* = 2.82, *p* = 0.005,95% CI [0.07,0.38]), indicating that eighth graders outperformed fourth graders at both altitudes when DBP was low or mean. However, at high DBP, the grade effect was not significant (*b* = 0.16, SE = 0.10, *t* = 1.53, *p* = 0.126,95% CI [−0.05,0.37];*b* = −0.01, SE = 0.06, *t* = −0.13, *p* = 0.900,95% CI [−0.12,0.10]), suggesting no grade difference in WM (Figures 2F, G). At 4,200 m, no significant grade effects were found at any DBP level (low DBP: *b* = −0.09, SE = 0.07, *t* = −1.34, *p* = 0.180,95% CI [−0.23,0.04]; mean DBP: *b* = −0.08, SE = 0.06, *t* = −1.40, *p* = 0.162,95% CI [−0.19,0.03]; high DBP: *b* = −0.07, SE = 0.09, *t* = −0.73, *p* = 0.467,95% CI [−0.25,0.11]), indicating that WM did not differ between grades regardless of DBP level (Figure 2H).

## Discussion

This study examined Verbal WM performance in fourth- and eighth-grade children at three altitudes (2,200, 3,200, and 4,200 m) to explore the developmental characteristics and relevant physiological correlates of WM in school-aged children exposed to altitudinal hypoxia. Results showed significant main effects of altitude on WM, as well as a significant grade-by-altitude interaction. Model comparison favored the developmental trajectory restructuring model over the simple additive model [LRT:χ2(2)=10.77,p=0.005]. Analysis of DBP also revealed a significant grade-by-altitude interaction. Further moderated regression analysis indicated a significant three-way interaction among grade, DBP, and altitude, suggesting that the moderating effect of DBP on WM development is altitude-dependent. Simple slope analysis showed that at 2,200 and 3,200 m, eighth-grade students outperformed fourth-grade students in WM only when DBP was at low or moderate levels; at 4,200 m, no significant grade differences were observed regardless of DBP level.

It is plausible that high-altitude hypoxia is associated with an altered developmental trajectory of WM, which is in line with the developmental trajectory reshaping model. At 2,200 m, eighth-grade students showed significantly better WM performance than fourth-grade students, reflecting typical cognitive developmental gains during school age; at 3,200 and 4,200 m, however, this grade-related cognitive advantage disappeared. We speculate that high-altitude hypoxia does not simply shift the developmental curve downward in a parallel fashion; rather, it may alter the normative developmental trajectory of WM. This alteration could arise from the following potential interpretations.

One possibility is a compression of the upper limit of cognitive development. Hypoxia may restrict the ultimate developmental ceiling of WM capacity, causing the developmental process to enter a plateau phase at an earlier age. That is, the cognitive development of children at high altitudes may reach a ceiling earlier, limiting further age-related cognitive gains and ultimately resulting in the absence of a significant performance advantage in higher-grade over lower-grade students. This interpretation is indirectly supported by prior empirical studies. Animal studies have shown that early-life hypoxia is associated with subsequent impairments in WM function (Golan et al., 2009; Wei et al., 2016). Meanwhile, both human cohort studies and animal experiments have indicated that early-life hypoxia and other forms of adversity may be associated with altered developmental maturation (Kaufman and Charney, 2001; Mukandala et al., 2016; Nalivaeva et al., 2018).

Another possibility is the delay of critical developmental periods. Prolonged hypoxia exposure may delay the maturation of WM. Longitudinal studies on infants and toddlers at high altitudes have indirectly supported this possibility by showing that prolonged exposure leads to persistent delays in gross motor development. This effect remained significant after controlling for confounding factors such as nutrition and family environment, highlighting the central role of hypoxia itself (Zhou et al., 2026). In low-altitude populations, the period from school age through adolescence represents a critical phase of rapid and fine-grained prefrontal cortex development (Casey et al., 2005). Sustained hypoxia may delay this critical window, which correlates with a rightward shift of the cognitive developmental trajectory in children. As a result, the accelerated phase of cognitive growth appears to be delayed among children living at high altitudes.

The third possibility is reversible inhibition of cognitive expression rather than impairment of capacity itself. Hypoxia may trigger adaptive strategies involving the reallocation of cerebral resources. Specifically, the brain prioritizes limited metabolic resources toward maintaining basic physiological homeostasis and fundamental cognitive operations, which may be linked to temporary suppression of higher-order cognitive processing. Evidence supporting this comes from multiple studies. Under acute hypoxia, simple WM remains largely unaffected, whereas performance on complex WM tasks is significantly impaired (Wang et al., 2022). Healthy young adults who had migrated to high altitudes for three years showed significantly decreased accuracy and increased reaction times on high-load verbal and spatial 2-back tasks. In contrast, their performance on low-load 1-back tasks did not differ significantly from baseline (Ma et al., 2019). Studies on native Tibetan residents have also revealed a similar functional selectivity. Spatial WM, which is closely tied to survival, remains intact, whereas verbal WM accuracy shows significant decline under chronic hypoxia (Li et al., 2026). Extrapolating these findings from adults to children suggests that high-altitude children may have WM capacity similar to their low-altitude counterparts. However, they may struggle to fully express this capacity behaviorally under hypoxic stress. If this inference holds, returning to low altitudes and reoxygenation could contribute to significant recovery in WM performance. A recent study provides indirect support for this possibility. Among native Tibetans who migrated to low altitudes, verbal WM progressively improved with prolonged reoxygenation, reaching baseline levels after three years (Li et al., 2026). This further suggests that the same inhibitory pathways may underlie WM performance in high-altitude children. If so, functional recovery following reoxygenation may also be achievable.

In addition, this study identified a tentative altitudinal trend in WM performance among school-aged children, suggesting a potential turning point somewhere between 3,200 and 4,200 m. WM performance in the 4,200 m group was significantly lower than in the 2,200 and 3,200 m groups for both fourth- and eighth-grade students, while no significant difference was observed between the two lower altitude groups. This finding is broadly consistent with studies on cognitive function in adults at high altitudes. For instance, studies at altitudes around 3,680 m have shown that migrants exhibit WM impairments (Ma et al., 2019), and native Tibetans show reversible deficits in verbal WM (Li et al., 2026). Similarly, in Han Chinese adults born and raised between 2,616 and 4,200 m, spatial WM was maintained through neural compensation, whereas verbal WM was directly impaired (Yan X. A. et al., 2011) These findings suggest that WM alterations begin to emerge at altitudes around 3,000 m or higher. It is speculated that neural compensatory capacity may tend to decline within this altitude range, and cognitive performance may show a downward shift above these elevations (Lefferts et al., 2019; Ma H.-L. et al., 2020; Williams et al., 2019). Cognitive behavioral evidence further supports this observation. At altitudes above 4,000 m, cerebral oxygen delivery declines markedly, which may ultimately lead to reduced WM performance (Bakker et al., 2023; Bliemsrieder et al., 2022; Dykiert et al., 2010).

Further analysis revealed that DBP moderates WM development in children at high altitude, and this moderating effect is altitude-dependent. This finding offers a physiological perspective for interpreting how DBP plays a moderating role in the altitude-dependent modulation of WM developmental changes. At altitudes of 2,200 and 3,200 m, eighth-grade students outperformed fourth-grade students in WM, but only among those with low or moderate DBP levels. In contrast, this grade-related advantage was absent in the high DBP group. Elevated BP is known to be one of the body’s compensatory responses to hypoxia and serves as an external indicator of physiological stress load (Lang et al., 2021). Accordingly, individuals with low or moderate DBP show a relatively mild physiological response to hypoxia, meeting cerebral oxygen demands without significantly increasing peripheral vascular resistance (Querido et al., 2011; Rao et al., 2015; Whayne, 2014). One interpretation is that their physiological stress load is low and their bodies remain in a relatively stable state. We speculate that their cognitive systems are less burdened by additional physiological pressure, allowing them to fully manifest the developmental advantages associated with increasing age.

In contrast, the high DBP group showed a different response pattern. Substantially elevated DBP typically reflects increased peripheral vascular resistance (Folkow, 1971) and indicates an excessive compensatory response to hypoxia, along with a higher physiological stress load (Lang et al., 2021). Sustained high DBP may reflect a less efficient compensatory response to hypoxia. Prior studies in adults have shown that altered blood pressure regulation under hypoxic conditions is associated with reduced cognitive performance (Bloomfield et al., 2022; Friend et al., 2019; Kang et al., 2025). This association raises the possibility that high DBP may be linked to a reduced ability of eighth-grade students to fully realize their developmental advantages, thereby obscuring the otherwise expected grade-related cognitive advantage. In other words, high stress load could be associated with altered cognitive development.

When altitude increased to 4,200 m, no significant grade differences in WM were observed regardless of DBP level, indicating that the moderating effect of DBP was almost completely neutralized. In other words, eighth-grade students showed a conditional advantage at 2,200 and 3,200 m, yet this advantage disappeared at 4,200 m. This finding suggests that under substantial hypoxia at 4,200 m, the physiological regulatory basis supporting age-related advantages appears to undergo a shift: the cognitive system prioritizes maintaining basic function over supporting developmental gains. Similar to prior adult research, our results suggest that WM performance tends to decrease notably within the altitude range of 3,200–4,200 m (Bliemsrieder et al., 2022; Ma H.-L. et al., 2020). Within this range, even when the body initiates compensatory mechanisms such as BP regulation, it remains difficult to meet the energy demands required for high-metabolic cognitive tasks, including WM (Kang et al., 2025). Ultimately, at higher altitudes within this range, the cognitive gains that typically accompany increasing age fail to materialize, and eighth-grade students no longer show a cognitive advantage.

Cross-sectional comparisons between fourth- and eighth-grade students can reveal developmental differences; however, they cannot track individual developmental trajectories or identify critical turning points. Expanding the age range, establishing normative data for children, and including a low-altitude control group or making comparisons with normative developmental curves would contribute to a more comprehensive understanding of the dynamic effects of high-altitude hypoxia on WM development in school-aged children. Future research may adopt longitudinal designs to better capture these developmental processes.

## Conclusion

High-altitude hypoxia alters the developmental trajectory of verbal WM in Tibetan school-aged children, as supported by model comparison favoring the developmental trajectory restructuring model over the simple additive model. The typical grade-related improvement in WM was evident at 2,200 m but disappeared at higher elevations, with a potential turning point between 3,200 and 4,200 m. DBP exerted an altitude-dependent moderating effect: high DBP weakened the developmental advantage at moderate to high altitudes, whereas this regulatory effect was completely lost at 4,200 m. These findings suggest that high-altitude hypoxia influences normal WM development, and the altitude-dependent modulation of DBP provides physiological evidence supporting this developmental alteration. These findings provide translational implications for neurocognitive screening, physical health monitoring, and targeted cognitive support strategies for children residing at high altitudes.

## Data Availability

The raw data supporting the conclusions of this article will be made available by the authors, without undue reservation.
