# Supplementary material for: High-altitude hypoxia alters the developmental trajectory of working memory in Tibetan children
Source: Front Neurosci. 2026 Jul 22;20:1843212. doi: 10.3389/fnins.2026.1843212 (PMC13437779; doi:10.3389/fnins.2026.1843212)
Supplement: Supplementary file 1 [file Data_Sheet_1.docx]

| Supplementary Table 1 Moderating Effects of Blood Pressure (SBP and DBP) and Altitude on Working Memory | | | | | | |
| --- | --- | --- | --- | --- | --- | --- |
| Outcome Variable | Predictor Variables | *b* | *SE* | t | p | 95% CI |
| WM | constant | 4.35 | 0.36 | 11.95 | 0.000 | (3.63,5.06) |
|  | Grade | 0.06 | 0.06 | 0.94 | 0.348 | (-0.07,0.19) |
|  | SBP | 0.04 | 0.03 | 1.09 | 0.276 | (-0.03,0.10 ) |
|  | Altitude range1(Z1) | -0.36 | 0.53 | -0.69 | 0.493 | (-1.40,0.67) |
|  | Altitude range2(Z2) | 0.21 | 0.53 | 0.39 | 0.698 | (-0.84,1.25) |
|  | Grade×SBP | -0.00 | 0.01 | -0.82 | 0.411 | (-0.01,0.01) |
|  | Grade×Altitude range1(Z1) | 0.08 | 0.09 | 0.94 | 0.345 | (-0.09,0.25) |
|  | Grade×Altitude range2(Z2) | -0.18 | 0.09 | -2.03 | 0.043 | (-0.35,-0.01) |
|  | SBP×Altitude range1(Z1) | -0.02 | 0.05 | -0.51 | 0.614 | (-0.12,0.07) |
|  | SBP×Altitude range2(Z2) | 0.02 | 0.04 | 0.43 | 0.667 | (-0.07,0.11) |
|  | Grade×SBP×Altitude range1(Z1） | 0.00 | 0.01 | 0.36 | 0.717 | (-0.01,0.02) |
|  | Grade×SBP×Altitude range2(Z2) | -0.00 | 0.01 | -0.57 | 0.566 | (-0.02,0.01) |
| WM | constant | 3.41 | 0.45 | 7.54 | 0.000 | (-2.52,4.30) |
|  | Grade | 0.22 | 0.08 | 2.82 | 0.005 | (0.07,0.38) |
|  | DBP | 0.12 | 0.04 | 3.19 | 0.002 | (0.05,0.19) |
|  | Altitude range1(Z1) | 0.52 | 0.58 | 0.91 | 0.365 | (-0.61,1.66) |
|  | Altitude range2(Z2) | 0.86 | 0.58 | 1.48 | 0.140 | (-0.28,2.00) |
|  | Grade×DBP | -0.02 | 0.01 | -3.08 | 0.002 | (-0.03,-0.01) |
|  | Grade×Altitude range1(Z1) | -0.06 | 0.10 | -0.62 | 0.539 | (-0.25,0.13) |
|  | Grade×Altitude range2(Z2) | -0.30 | 0.10 | -3.10 | 0.002 | (-0.49,-0.11) |
|  | DBP×Altitude range1(Z1) | -0.10 | 0.05 | -1.97 | 0.050 | (-0.20,0.00) |
|  | DBP×Altitude range2(Z2) | -0.12 | 0.05 | -2.65 | 0.008 | (-0.21,-0.03) |
|  | Grade×DBP×Altitude range1(Z1） | 0.02 | 0.01 | 2.28 | 0.023 | (0.00,0.03) |
|  | Grade×DBP×Altitude range2(Z2) | 0.02 | 0.01 | 2.56 | 0.011 | (0.00,0.03) |
| Note: Altitude was dummy-coded with 3,200 m as the reference group. Z1 compared 2,200 m vs. 3,200 m; Z2 compared 4,200 m vs. 3,200 m. | | | | | | |
